# Supplementary material for: Choice-based severity scale (CSS): assessing the relative severity of procedures from a laboratory animal’s perspective
Source: PeerJ. 2024 Jun 17;12:e17300. doi: 10.7717/peerj.17300 (PMC11188928; doi:10.7717/peerj.17300)
Supplement: Supplemental Information 1 [file peerj-12-17300-s001.docx]

Supplementary material for: ‘Choice-based Severity Scale (CSS): Assessing the relative severity of procedures from a laboratory animal’s perspective’

Lauren C. Cassidy, Stefan Treue, Alexander Gail, and Dana Pfefferle

# Guidelines for Choice-based Severity Assessments in animals

First, it is essential that the animals know what the conditions are and are associated with representative stimuli properly. In our study, we initially trained the monkeys to associate visual stimuli with a small motivational reward from the cage condition cognitive testing system (cage condition) or once seated in the non-human primate chair (laboratory condition). During training, each condition stimulus was paired with an unrewarded stimulus for several trials each day. Training was repeated until the monkeys learned to select the condition stimuli at least 75 % of trials per trial type in multiple sessions.

Second, it is important to ensure that the experimental setup where the animals are given choices minimizes arousal and influence from the experimenter ([Schmitt, Schloegl & Fischer, 2014](#ref-schmitt_seeing_2014)), which could confound animals’ choices, and is unbiased. By placing the experimental setup close to the monkeys’ home cage, we minimized arousal by eliminating the need for additional transport ([Habedank et al., 2018](#ref-habedank_severity_2018); [Kahnau et al., 2022](#ref-kahnau_determining_2022)). Additionally, this setup took advantage of the existing infrastructure and did not require a separate room for testing. We did, however, need to conduct several pilot experiments to reduce environmental bias (see [‘Supplementary experiments’](#X0b8095901bde5d5f6af66fbbb3a893024f3157e)). Additionally, we tested two compartment positions of the cage condition (position on the upper and lower quadrants of the compartment) to control that choices were made for the condition rather than the monkeys’ natural tendency to go up ([Clarence et al., 2006](#ref-clarence_use_2006)).

Third, an animals’ prior experience with the conditions provided may have an influence on their choice behavior ([Arnold & Estep, 1994](#ref-arnold_laboratory_1994); [Fraser & Nicol, 2011](#ref-fraser_preference_2011); [Habedank et al., 2018](#ref-habedank_severity_2018)). Supp. Table 1 provides more detail about the monkeys’ experiences in our study. Preferences are difficult to interpret if one option is more familiar than the other as they could be due to novelty seeking or risk aversion ([Dawkins, 1977](#ref-dawkins_hens_1977); [Habedank, Kahnau & Lewejohann, 2021](#ref-habedank_alternate_2021)). Ideally, animals would have equal experience with all conditions, but such a goal is often impractical in research settings. Moreover, the animals experience with different conditions is arguably what shapes and renders their subjective perspective. Therefore, we emphasize the importance of sufficient general and recent familiarity before Choice-based Severity Assessments. For example, our monkeys were extensively trained in both the cage and laboratory conditions prior to our study and the CSS protocol ensured that their most recent experiences were balanced between the two conditions. Importantly, none of the monkeys had chronic implants at the time of testing and only one animal had such an experience previously. Chronic implants require regular inspection and treatment to prevent the development of issues, such as infection, which can cause pain. Therefore, a monkey with these past aversive experiences in association to the laboratory condition may be less willing—-and have a stronger aversion—-to participate in related procedures despite no implant treatment taking place.

# Supplementary information

## Additional study subject information

**Supp. Table 1: Additional information about the study subjects and their experience.** Laboratory is abbreviated as “lab”.

| **Monkey** | **Origin** | **Age** | **Implanted** | **Task** | **Age entered lab** | **Chair training** | **Lab condition training** | **Preferred juice** |
| --- | --- | --- | --- | --- | --- | --- | --- | --- |
| H | Germany | 11 yrs | Never | Random dot-pattern change | 4 yrs | 6 yrs | 5 yrs | Grape |
| D | China | 10 yrs | Never | Stimulus color change | 5 yrs | 4 yrs | 3 yrs | Banana |
| E | Germany | 19 yrs | Explanted | Random dot-pattern change | 3 yrs | 15 yrs | 15 yrs | Grape |

## Preparation for the Choice-based Severity Assessment

### Basic experimental task training

Prior to conducting the Choice-based Severity Assessment, we trained all monkeys to perform a basic experimental task commonly used as a first training step in neuroscience laboratories (i.e., touch-hold-release). The monkeys learned to perform either a stimulus color (monkey D) or random dot-pattern direction change (monkeys H and E) task, that could be carried out by holding and releasing a sensor in either the cage or laboratory condition. Both basic experimental tasks are described in detail in the next sections. During the training phase, the monkeys were rewarded with 0.3 ml (one drop) of grape juice for each correct trial (i.e., detecting a change in the stimulus). Prior to any choice testing, we ensured that the monkeys could perform the basic experimental task successfully over 80 % of trials within a session. Training sessions ran for two hours in both cage and laboratory conditions. From this training data, we examined the level of engagement (i.e., when trials were being conducted) across the two hours of training for multiple training sessions in each condition, as the monkeys often conducted most trials for a session within the first hour of training. Then we determined an inter-trial interval for each monkey that would generally indicate that it had completed at least 80 % of trials for these 2-hour training sessions in both conditions (monkey H: 12 min; monkey D: 9 min; monkey E: 8 min). We used this inter-trial interval as an indicator that the monkey was not interested in engaging in the basic experimental task further. This conclusion criterion was important to apply as the monkeys may have viewed excess time spent in either condition as a punishment. The basic experimental task was programmed to automatically stop once the conclusion criteria for the individual had been met, and the experimenter returned the monkeys to their home cage soon afterwards.

#### Stimulus color change task

In the stimulus color change task (monkey D), a green square (0.2 cm) was presented in the center of the screen for 2.5 s or until the monkey touched the sensor. If the sensor was not engaged, the stimulus disappeared and then reappeared again after 1.4 s. If the monkey engaged the sensor while no stimulus was present, the task emitted a soft beep until the monkey removed his hand. When the sensor was touched when the stimulus was present, the luminance of the square decreased and remained on the screen for a randomly selected duration between 0.4 and 2 s, as long as the monkey continued to hold the sensor. An ‘error’ sound occurred if the sensor was released early (i.e., early release), followed by a 1 s timeout. Once the randomly selected duration expired, then the square stimulus turned red and the monkey had to release the sensor within 2.5 s to receive a fluid reward and hear a ‘ding’ sound, indicating a correct trial. If the monkey continued to hold the sensor longer than the response window, then the stimulus disappeared and a ‘beep’ sound occurred every 1 s until the monkey released the sensor (i.e., late response). The basic steps of the stimulus color change task are depicted in steps two to four of Figure 3 of the main text.

#### Random dot-pattern direction change task

The basis of the random dot-pattern direction change task (monkeys H and E) was similar the stimulus color change task in that the monkeys was required to respond to a change in a random dot pattern stimulus via a sensor. In this task, a soft ‘beep’ sound occurred every 2.5 s until the monkey initiated a trial by touching the sensor. Immediately after the monkey touched the sensor, a random dot pattern stimulus appeared in the center of the screen (dot size: 0.01 cm in diameter; dot density: 3.54 dots per cm^2^; speed: 5 cm per s) moving in one direction (of 360 degrees) for a randomly chosen duration between 0.3 and 2.5 s (monkey H: 0.3 to 2.5 s; monkey E: 0.3 to 1.2 s). After the randomly chosen duration expired, the direction of the stimulus changed by 45 degrees clockwise or counterclockwise randomly and the monkey had to respond to the change by releasing the sensor within 0.7 s. An ‘error’ sound and a 1 s timeout occurred if the monkey released the sensor early. If the monkey continued to hold the sensor past the expiration of the response window, then a ‘beep’ sound occurred every 2 s until the monkey released the sensor (i.e., late response). The basic steps of the random dot-pattern direction change task are depicted in steps two to four of Figure 3 of the main text.

### Choice task and condition stimuli training

In addition to learning the basic experimental task, the monkeys were trained to engage with the choice task and condition stimuli that were used in the Choice-based Severity Assessment. Generally, the choice task was structured so that the monkey needed to initiate a trial by touching a small square (i.e., start button) appearing in a black box within a colored bar spanning the lower third of the touchscreen of the neutral cognitive testing system. The monkey needed to touch the start button within 5 s for the condition stimuli to be presented, ensuring his attention was directed towards the touchscreen, otherwise the start button disappeared.

Condition stimuli were introduced in two stages. During the familiarization stage, each condition stimulus appeared on the left or right of the touchscreen on its own for 3 s or until touched by the monkey (a 1 to 10 s timeout and ‘error’ sound occurred if stimulus was not touched). Each condition stimulus constituted a 6.6 cm by 6.6 cm picture (laboratory condition: non-human primate chair; cage condition: cognitive testing system with the cage condition stimulus on the touchscreen) and a 11 cm by 11 cm colored background specific for the condition (colors differed between monkeys). Once the monkeys touched the presented condition stimulus, a ‘ding’ sound was produced and the stimulus deluminated for 0.75 s before disappearing. The sliding panel of the corresponding quadrant in the testing compartment was opened and closed by the experimenter once the monkey had entered. If the cage condition stimulus was selected, the same condition stimulus appeared on the cage condition cognitive testing system, which the monkey triggered to disappear (by either touching the touchscreen or using the proximity sensor) to dispense a small motivational reward (i.e., 2 ml bolus of water). If the laboratory condition stimulus was selected, the experimenter opened the cage door to the non-human primate chair so that the monkey could climb in and receive the 2 ml bolus once seated. The laboratory condition stimulus was mounted on a larger (same) colored background to the front of the non-human primate chair so that the monkey could see the stimulus as he entered the chair. After each familiarization training trial, the monkey was returned to the quadrant with the neutral cognitive testing system. The procedure for the differentiation stage of condition stimulus training was the same except that a stimulus associated with a short timeout (i.e., timeout stimulus) was presented simultaneously on the opposite side of the touchscreen. The timeout stimulus was introduced to train the monkey to make an informed choice. If the timeout stimulus was touched, both stimuli disappeared, and an ‘error’ sound was produced. Otherwise if the condition stimulus was touched, the timeout stimulus disappeared immediately and the condition stimulus deluminated for 0.75 s before disappearing.

In both stages, the position and type of stimulus was counterbalanced every four trials. Training sessions generally consisted of 24 trials, counterbalanced by condition stimulus type. Training ended after the monkeys chose the condition stimulus over the timeout stimulus for at least 75 % of trials for each trial type in a session.

### Fluid preference test

Prior to the third phase, we tested the relative fluid reward preferences of each monkey. We presented five different types of fluid rewards simultaneously once a day for 10 min to each monkey, counterbalancing the position of each option across five days. Monkey E was tested using different fluid rewards than monkeys D and H due to dietary restrictions. We used the highest mean amount (ml) of fluid consumed as the monkey’s preferred fluid reward (monkeys H and E: grape juice; monkey D: banana juice). As preferences may shift over time, reward preferences should be regularly evaluated.

## Preparation of the Choice-based Severity Scale test

During the Choice-based Severity Scale test, the monkeys were given a choice between two tasks differing in the duration of how long they had to hold a proximity sensor (i.e., ‘sensor’), until they detected a change in the task stimulus (i.e., short and long hold tasks; see Figure 5 in the main text). Short and long tasks were differentiated by colored stimuli of the same shape and adapted from the basic experimental task the monkey engaged with during the Choice-based Severity Assessment (see [‘Basic experimental task training’](#X72d87585e3fa632f31435cb472b1da7a7fe91e2)).

During training, we introduced the concept of making a choice between colored stimuli by repeatedly presenting one task stimulus with a timeout stimulus (that differed in color) simultaneously, with the position of the stimuli counterbalanced across trials. Over the course of training, we slowly increased the duration of the hold of each task until it reached its corresponding hold range. Specifically, monkey D was trained to hold the short hold task for a randomized duration between 1 and 4 s, whereas the hold for the long hold task was between 10 s and 40 s. Monkeys H and E were trained on shorter hold durations due to time constraints, but these durations still differed in their range by a factor of 10 (short hold: 1 to 3 s; long hold: 10 to 30 s). We added abort penalty so that the monkeys were encouraged to complete trials of both tasks and receive the corresponding fluid reward. The duration of the abort penalty corresponded to the average hold duration (short hold: 2 to 2.5 s; long hold: 20 to 25 s). We provided more reward (of the monkeys’ preferred juice) for long hold trials during training to maintain the interest, compensate for the additional hold duration, and to keep the reward per second roughly equivalent between the two tasks. At the end of training, the difference in reward between the two tasks was 2 or 3 ml, with a payout of approximately 0.11 to 0.13 ml per s of hold (short hold: 0.3 ml for monkey D, 0.2 ml for monkeys H and E; long hold: 3.3 ml for monkey D, 2.2 ml for monkeys H and E). Training sessions were two hours in length, where each task was presented with the timeout stimulus for approximately one hour each (order alternated each session). We checked that the monkeys could perform both tasks successfully (over 80 % correct for each task within a session) prior to testing.

# Supplementary results

## Results of the Choice-based Severity Assessment for the third monkey (E)

We offered a third monkey (E) a choice between performing a basic experimental task in the lower cage or laboratory condition. Monkey E was only tested on the third phase, where the type of reward was different between the two conditions (cage: water; lab: grape juice). Like monkey D, monkey E exclusively chose the cage condition (100 % of six choice sessions) despite the amount and type of reward per trial being largely in favor of the laboratory condition (Supp. Figure 1).

**Supp. Figure 1: Results of the Choice-based Severity Assessment with monkey E.** Proportions were calculated using three consecutive choice sessions (represented by point size) as we assessed preference and adjusted the reward per trial difference accordingly at this time. The reward per trial difference is indicated by color. One bout of testing occurred over nine days due to one reference session per condition occurring prior to for each choice session. Laboratory is abbreviated as “lab”.

## Model description and results of the condition stimuli training for the Choice-based Severity Assessment

We trained the monkeys to associate a visual stimulus to either receiving a 2 ml bolus from the cage condition cognitive testing system (cage condition) or once they were seated in the non-human primate chair (laboratory condition) prior to the Choice-based Severity Assessment (the ‘Choice’ and ‘Initial bolus’ panels of the reference sessions in Figure 2 of the main text depict the basic training steps). During training, each condition stimulus was paired with a timeout stimulus multiple times over a session for each monkey. Therefore, we fit three GLMMs (one per monkey) with the response variable as the number of correct responses (i.e., touching the condition stimulus) in relation to the total number of trials tested of each trial type for each session. Trial type was our main fixed effect of interest. We added session number as an additional fixed effect and date tested as a random effect, with all possible random slopes to allow the slopes to vary across sessions ([Schielzeth & Forstmeier, 2009](#ref-schielzeth_conclusions_2009); [Barr et al., 2013](#ref-barr_random_2013)).

There was substantial evidence that all monkeys were able to differentiate the condition stimuli from the unrewarded stimulus, where monkey D also performed substantially better for upper cage than laboratory condition stimulus trials (Supp. Table 2; Supp. Figure 2). Generally, performance on cage condition trials was higher on average than laboratory condition stimulus trials (monkey H, upper cage: 0.99 % ± 0.02 %; monkey H, lab: 0.72 % ± 0.31 %; monkey D, cage: 1.00 % ± 0.01 %; monkey D, lab: 0.78 % ± 0.08 %; monkey E, lower cage: 0.96 % ± 0.10 %; monkey E, lab: 0.82 % ± 0.19 %). Overall, these results validate that the monkeys understood the link between the visual stimuli and their associated conditions during the Choice-based Severity Assessment. Additionally, these findings suggest that the monkeys preferred to receive a reward from the cage condition cognitive testing system than in the non-human primate chair associated with the laboratory condition. Such preferences may be driven by the differences in movement constraint between the two training trial types. Given that movement constraint is the first of other expected costs to laboratory condition (i.e., sociality, transport; see Figure 1 and Table 1 in main text), preferences for the cage condition during the Choice-based Severity Assessment would be expected.

**Supp. Figure 2: Results of the condition stimuli training for the Choice-based Severity Assessment.** The light grey points indicate the proportion of trials each condition stimulus was chosen (i.e., correct response) over the total number of trials conducted for that stimulus type for each monkey (N = 3) and session they participated in (range: 2 to 44 trials). The black points indicate the model probability estimates for each trial type. Whiskers represent the 95 % credible intervals. Laboratory is abbreviated as “lab”.

**Supp. Table 2: Model results of the condition stimuli training for the Choice-based Severity Assessment.** Binomial generalized linear mixed models (one per monkey) tested whether the correct stimulus was chosen (i.e., condition stimulus).

| **Monkey** | **Variable** | **Estimate** | **SD** | **Lower CI** | **Upper CI** | **Pr** |
| --- | --- | --- | --- | --- | --- | --- |
| H | Intercept | 1.28 | 0.59 | 0.02 | 2.37 | 0.98 |
|  | Trial type (Upper cage)^a^ | 0.9 | 0.57 | -0.25 | 1.93 | 0.94 |
|  | Trial number | 0.07 | 0.35 | -0.66 | 0.71 | 0.60 |
| D | Intercept | 1.31 | 0.12 | 1.07 | 1.56 | 1.00 |
|  | Trial type (Upper cage)^a^ | 2.04 | 0.33 | 1.41 | 2.65 | 1.00 |
|  | Trial number | 0.02 | 0.12 | -0.22 | 0.25 | 0.55 |
| E | Intercept | 1.81 | 0.38 | 0.98 | 2.56 | 1.00 |
|  | Trial type (Lower cage)^a^ | 0.5 | 0.44 | -0.4 | 1.35 | 0.87 |
|  | Trial number | -0.68 | 0.3 | -1.28 | -0.08 | 0.98 |
| Estimate: slope of the predictor; SD: standard deviation of the estimate; CI: 95 % credible interval; Pr: proportion of the posterior samples that fall on the same side of 0 as the mean. ^a^The laboratory condition was the reference level for trial type. | | | | | | |

# Supplementary experiments

**Supp. Table 3: Compilation of the pilot experiments that informed the design of the Choice-based Severity Assessment.** Laboratory is abbreviated as “lab”.

| **Choice-based Severity Assessment: Vertically positioned conditions** | | **Result figure(s)** | | | **Main conclusions** |
| --- | --- | --- | --- | --- | --- |
| Purpose: First testing structure | 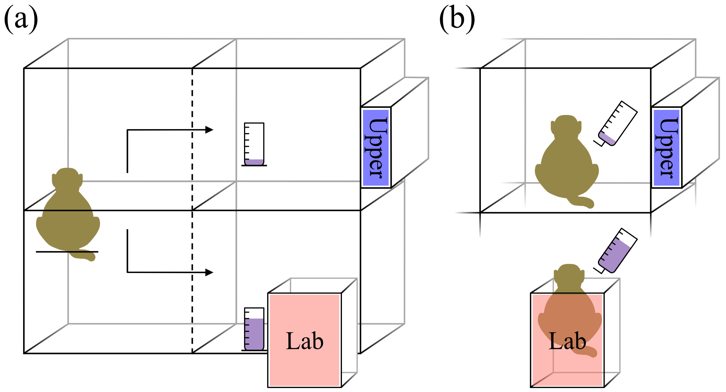  (a) Positioning of the conditions and their associated bolus as indicated by the cylinders. The vertical sliding panels were pulled simultaneously so the monkey could choose a condition by entering one of the quadrants. (b) Once the monkey made a choice, it received the corresponding bolus of the chosen condition. |   Plot of monkey’s choices across sessions. The bolus associated with each condition was adjusted according to the monkey’s choice using two interleaved staircases that alternated each session. | | - The monkey only chose the cage condition. - Unclear if this preference was due to the position or the option itself. - The monkey also might not have paid attention to or been able to differentiate the volumes of fluid. | |
| **Choice-based Severity Assessment: Volume differentiation training** | | **Result figure(s)** | | | **Main conclusions** |
| Purpose: To train the monkeys to pay attention to the fluid reward cylinders and to determine their volume differentiation skills. | 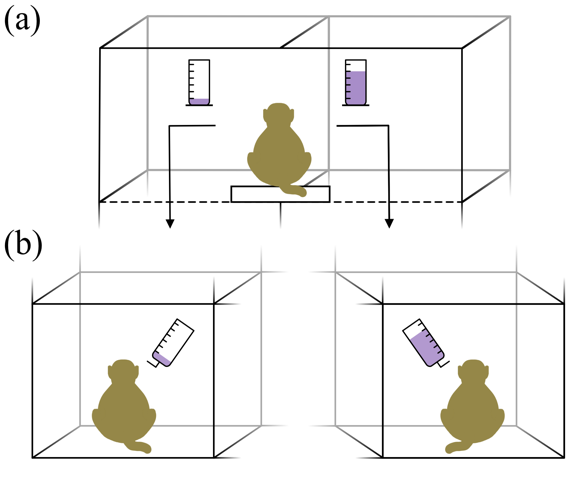  (a) Once the monkey was centered between the top two quadrants, cylinders with different amounts of bolus were placed on either side for the monkey to choose between. The monkey indicated his choice by tapping the position of one of the cylinders. (b) Once the monkey made a choice, the horizontal sliding panel was opened to the corresponding quadrant. The monkey received the chosen bolus once he entered the open lower quadrant for both conditions. |   Proportion of trials that the monkeys’ chose the cylinder with the larger fluid reward volume. The pairs of volumes presented were 0 vs. 60, 5 vs. 55, 10 vs. 50, 15 vs. 45, 20 vs. 40, and 25 vs. 35. 30 vs. 30 was also tested by is not presented in the graph. | - The monkeys were quite good at differentiating volumes (choosing the larger volume over 75% of trials) when the difference between the cylinders was over 25 ml. - This training was conducted in preparation for the next pilot choice experiment (horizontally positioned conditions). | | |
| **Choice-based Severity Assessment: Horizontally positioned conditions** | | **Result figure(s)** | | | **Main conclusions** |
| Purpose: To control for the bias due to positioning the options up and down | 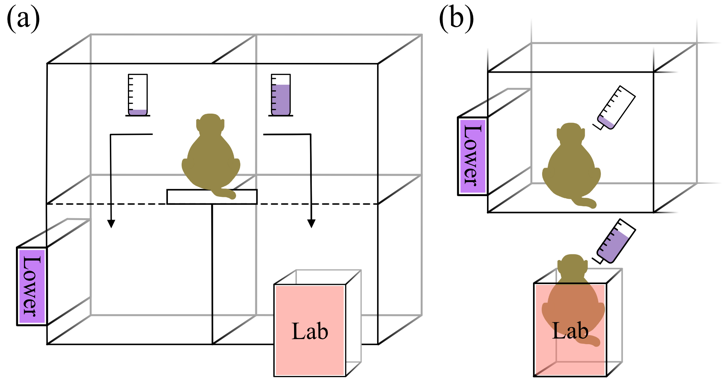  (a) Positioning of the conditions and their associated bolus as indicated by the cylinders. Once the cylinders were placed into position, the monkey indicated his choice by tapping the position of one of the cylinders. (b) Once the monkey made a choice, the horizontal sliding panel was opened to the corresponding quadrant. The monkey received the chosen bolus once he had moved into the open lower quadrant. |   (a) Proportion of sessions that the lab condition was chosen per reward difference for each monkey. (b) Number of sessions that the monkeys chose each condition by their position during the choice phase of the experiment. | | | - We can influence the monkeys’ choice with reward (psychometric function graph). - Monkeys exhibited a side bias (independent of the condition type). - Potentially due to the location of the cage squeeze (needed for veterinary purposes) on the lower right compartment. |
| **Choice-based Severity Assessment: Tunnel positioned conditions** | | **Result figure(s)** | | | **Main conclusions** |
| Purpose: To control for the bias due to positioning the options left and right. | 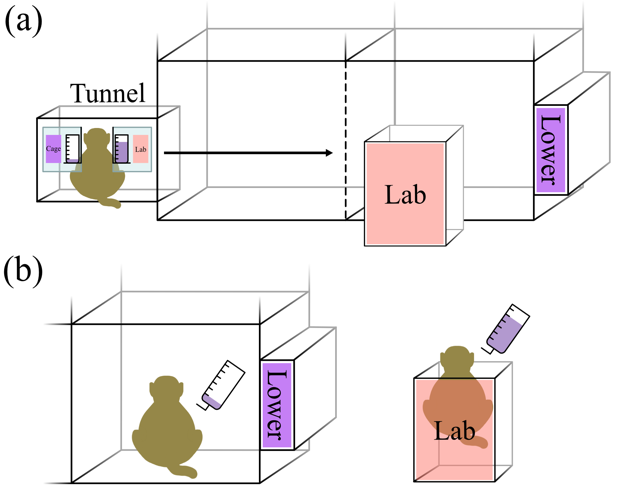  (a) Positioning of the conditions and location of the tunnel where the monkey made his choice. Once the condition tags and cylinders were placed into position, the monkey indicated his choice by tapping the position of the condition. (b) The bolus for the cage condition was received in the lower right quadrant once the non-human primate chair was removed. The bolus for the laboratory condition was received once the monkey had popped his head out of the non-human primate chair neck plate. | ****  (a) Number of sessions that the monkeys chose each condition by their position when offered a choice between conditions. (b) The proportion of sessions in the last six that were positioned on the left during the choice phase of the experiment. | | | - Monkeys still prone to side biases. - Might also not have understood that each condition stimulus and its corresponding bolus lead to different consequences. |
| **Choice-based Severity Scale test: Counterbalanced options** | | **Result figure(s)** | | | **Main conclusions** |
| Purpose: First testing structure of the Choice-based Severity Scale test. |  |   Proportion of trials that the monkeys chose the option positioned on the left per session. | | | - One individual developed a strong side bias by the second session. - Result suggested that the monkeys could respond without an informed decision. - Therefore, we changed the position of the options to be deterministic (see main text). |
| Note: For all pilot experiments where the monkeys were offered a choice between performing the basic experimental task in the cage and laboratory conditions, the task was started 10 minutes later to account for the time needed to transport the monkey to the laboratory condition. The basic experimental task ran for 2 hours in both conditions. Contact the first-author of the study for more specific information on how the pilot experiments were carried out. | | | | | |

# Supplementary video

See video in a separate file.

# Supplementary references

Arnold CE, Estep DQ. 1994. Laboratory caging preferences in golden hamsters (*Mesocricetus auratus*). *Laboratory Animals* 28:232–238. DOI: [10.1258/002367794780681598](https://doi.org/10.1258/002367794780681598).

Barr DJ, Levy R, Scheepers C, Tily HJ. 2013. Random effects structure for confirmatory hypothesis testing: Keep it maximal. *Journal of Memory and Language* 68:255–278. DOI: [10.1016/j.jml.2012.11.001](https://doi.org/10.1016/j.jml.2012.11.001).

Clarence WM, Scott JP, Dorris MC, Paré M. 2006. Use of Enclosures with Functional Vertical Space by Captive Rhesus Monkeys (Macaca mulatta) Involved in Biomedical Research. *Journal of the American Association for Laboratory Animal Science* 45:4.

Dawkins M. 1977. Do hens suffer in battery cages? Environmental preferences and welfare. *Animal Behaviour* 25:1034–1046. DOI: [10.1016/0003-3472(77)90054-9](https://doi.org/10.1016/0003-3472(77)90054-9).

Fraser D, Nicol CJ. 2011. Preference and motivation research. *Animal welfare*:183–199. DOI: [10.1079/9781845936594.0183](https://doi.org/10.1079/9781845936594.0183).

Habedank A, Kahnau P, Diederich K, Lewejohann L. 2018. [Severity assessment from an animal’s point of view](https://www.cabdirect.org/cabdirect/abstract/20183235031). *Berliner und Münchener Tierärztliche Wochenschrift* 131:304–320.

Habedank A, Kahnau P, Lewejohann L. 2021. Alternate without alternative: Neither preference nor learning explains behaviour of C57BL/6J mice in the T-maze. *Behaviour* 158:625–662. DOI: [10.1163/1568539X-bja10085](https://doi.org/10.1163/1568539X-bja10085).

Kahnau P, Jaap A, Diederich K, Gygax L, Rudeck J, Lewejohann L. 2022. Determining the value of preferred goods based on consumer demand in a home-cage based test for mice. *Behavior Research Methods*. DOI: [10.3758/s13428-022-01813-8](https://doi.org/10.3758/s13428-022-01813-8).

Schielzeth H, Forstmeier W. 2009. Conclusions beyond support: Overconfident estimates in mixed models. *Behavioral Ecology* 20:416–420. DOI: [10.1093/beheco/arn145](https://doi.org/10.1093/beheco/arn145).

Schmitt V, Schloegl C, Fischer J. 2014. Seeing the experimenter influences the response to pointing cues in long-tailed macaques. *PLoS ONE* 9:e91348. DOI: [10.1371/journal.pone.0091348](https://doi.org/10.1371/journal.pone.0091348).
