# Supplementary material for: Choice-based severity scale (CSS): assessing the relative severity of procedures from a laboratory animal’s perspective
Source: PeerJ. 2024 Jun 17;12:e17300. doi: 10.7717/peerj.17300 (PMC11188928; doi:10.7717/peerj.17300)
Supplement: Supplemental Information 4 [file peerj-12-17300-s004.zip › Analysis code and data/240219 Choice-based Severity Scale metadata.docx]

# Metadata for:

*Choice-based Severity Scale (CSS): Assessing the relative severity of procedures from a laboratory animal’s perspective*

Lauren C. Cassidy, Stefan Treue, Alexander Gail, and Dana Pfefferle

# Table 1: Variables, their definitions, and the levels or range of the variables for the condition stimuli training data (data set 1).

| **Variables** | **Definition** | **Levels/Range** |
| --- | --- | --- |
| monkeyID | Identity of the monkey being trained (data examined separately by monkey). | *monkeyD*: monkey D  *monkeyH*: monkey H  *monkeyE*: monkey E |
| testPhase | Experimental phase of the Choice-based Severity Assessment (factor). | *1*: choice between the upper cage and lab conditions (same reward type in task).  *2*: choice between the lower cage and lab conditions (same reward type in task).  *3*: lower cage condition vs. lab condition (different fluid type in task). |
| boutNum | Number of bouts that the monkey was tested (covariate). | Range: 1 to 10. |
| cond1 | The first of two conditions simultaneously presented to the monkey to choose between (factor). | *upperCage*: monkey moves to the upper cage to conduct the basic experimental task.  *lowerCage*: monkey moves to the lower cage to conduct the basic experimental task. |
| cond2 | The second of two conditions simultaneously presented to the monkey to choose between (factor). | *lab*: monkey is transported to the lab to conduct the basic experimental task. |
| cond1_rewardType | Type of reward received for correct trials during the basic experimental task of condition 1 (factor). | *grapeJuice*: correct trials were rewarded with grape juice.  *water*: correct trials were rewarded with water. |
| cond2_rewardType | Type of reward received for correct trials during the basic experimental task of condition 2 (factor). | *grapeJuice*: correct trials were rewarded with grape juice.  *bananaJuice*: correct trials were rewarded with banana juice. |
| rewardDifference | Difference in the number of drops of reward distributed for correct trials in the basic experimental task of each condition (factor). | *largelyFavorsLab*: 9 drops distributed for correct trials of the basic experimental task in the lab condition and 1 drop for correct trials of the same task in the cage condition.  *mildlyFavorsLab*: 7 vs. 3 drops.  *equallyRewarded*: 5 vs. 5 drops.  *mildlyFavorsCage*: 3 vs. 7 drops.  *largelyFavorsCage*: 1 vs. 9 drops. |
| cageCond_count | Number of times the monkey chose the cage condition in the bout of choice sessions (to calculate response). | Range: 0 to 3. |
| labCond_count | Number of times the monkey chose the lab condition in the bout of choice sessions (to calculate response). | Range: 0 to 3. |
| total | Total number of choice sessions completed within the bout (to calculate response). | Range: 1 to 3. |
| boutProp | Proportion of the bout of choice sessions (see “total”) that the lab condition was chosen (response). | Range: 0 to 1. |

# Table 2: Variables, their definitions, and the levels or range of the variables for the Choice-based Severity Scale test data (data set 2).

| **Variables** | **Definition** | **Levels/Range** |
| --- | --- | --- |
| dateTested | Date that the monkey was tested (random effect). | From 2022-04-25 to 2022-05-06 |
| monkeyID | Identity of the monkey being tested (data analyzed separately by monkey). | *monkeyD*: monkey D  *monkeyH*: monkey H  *monkeyE*: monkey E |
| sessionNum | Number of the session that the monkey was tested (covariate). | Range: 1 to 10. |
| trialNum | Number of the trial within a session (covariate). | Range: 1 to 391. |
| choicePosition | Position of selected option on the touchscreen (factor). | *left*: selected option positioned on the left of the touchscreen.  *right*: selected option positioned on the right of the touchscreen. |
| optionChosen | Option selected by the monkey at the beginning of the trial (response). | *shortHold*: the monkey had to hold a sensor between 1 and 4 s to receive 0.2 to 0.3 ml water reward.  *longHold*: the monkey had to hold a sensor between 10 and 40 s to receive 15.2 to 15.3 ml juice reward. |

# Table 3: Variables, their definitions, and the levels or range of the variables for the condition stimuli training data in preparation for the Choice-based Severity Assessment (data set 3).

| **Variables** | **Definition** | **Levels/Range** |
| --- | --- | --- |
| dateTested | Date that the monkey was trained (random effect). | From 2020-11-03 to 2021-09-30. |
| monkeyID | Identity of the monkey being trained (data analyzed separately by monkey). | *monkeyD*: monkey D  *monkeyH*: monkey H  *monkeyE*: monkey E |
| sessionNum | Number of the session that the trial type was trained (covariate). | Range: 1 to 27. |
| trialType | Type of stimuli presented to the monkey (factor). | *upperCage*: upper cage condition stimulus presented simultaneously with a timeout stimulus (for monkey D and H).  *lowerCage*: lowe rcage condition stimulus presented simultaneously with a timeout stimulus (for monkey E).  *lab*: lab condition stimulus presented simultaneously with a timeout stimulus (all monkeys). |
| correct | Number of correct trials completed by the monkey (response). | Range: 0 to 39. |
| total | Total number of trials completed by the monkey (response). | Range: 2 to 44. |
