# Supplementary material for: Choice-based severity scale (CSS): assessing the relative severity of procedures from a laboratory animal’s perspective
Source: PeerJ. 2024 Jun 17;12:e17300. doi: 10.7717/peerj.17300 (PMC11188928; doi:10.7717/peerj.17300)
Supplement: Supplemental Information 4 [file peerj-12-17300-s004.zip › Analysis code and data/240219 Choice-based Severity Scale metadata.pdf]

Metadata for:

Choice-based Severity Scale (CSS): Assessing the relative severity of procedures from a laboratory animal's perspective

Lauren C. Cassidy, Stefan Treue, Alexander Gail, and Dana Pfefferle

Table 1: Variables, their definitions, and the levels or range of the variables for the condition stimuli training data (data set 1).

Variables	Definition	Levels/Range
monkeyID	Identity of the monkey being trained (data examined separately by monkey).	*monkeyD*: monkey D<br>*monkeyH*: monkey H<br>*monkeyE*: monkey E
testPhase	Experimental phase of the Choice-based Severity Assessment (factor).	*1*: choice between the upper cage and lab conditions (same reward type in task).<br>*2*: choice between the lower cage and lab conditions (same reward type in task).<br>*3*: lower cage condition vs. lab condition (different fluid type in task).
boutNum	Number of bouts that the monkey was tested (covariate).	Range: 1 to 10.
cond1	The first of two conditions simultaneously presented to the monkey to choose between (factor).	*upperCage*: monkey moves to the upper cage to conduct the basic experimental task.<br>*lowerCage*: monkey moves to the lower cage to conduct the basic experimental task.
cond2	The second of two conditions simultaneously presented to the monkey to choose between (factor).	*lab*: monkey is transported to the lab to conduct the basic experimental task.
cond1_rewardType	Type of reward received for correct trials during the basic experimental task of condition 1 (factor).	*grapeJuice*: correct trials were rewarded with grape juice.<br>*water*: correct trials were rewarded with water.
cond2_rewardType	Type of reward received for correct trials during the basic experimental task of condition 2 (factor).	*grapeJuice*: correct trials were rewarded with grape juice.<br>*bananaJuice*: correct trials were rewarded with banana juice.
rewardDifference	Difference in the number of drops of reward distributed for correct	*largelyFavorsLab*: 9 drops distributed for correct trials of

Variables	Definition	Levels/Range
	trials in the basic experimental task of each condition (factor).	the basic experimental task in the lab condition and 1 drop for correct trials of the same task in the cage condition. *mildlyFavorsLab*: 7 vs. 3 drops. *equallyRewarded*: 5 vs. 5 drops. *mildlyFavorsCage*: 3 vs. 7 drops. *largelyFavorsCage*: 1 vs. 9 drops.
cageCond_count	Number of times the monkey chose the cage condition in the bout of choice sessions (to calculate response).	Range: 0 to 3.
labCond_count	Number of times the monkey chose the lab condition in the bout of choice sessions (to calculate response).	Range: 0 to 3.
total	Total number of choice sessions completed within the bout (to calculate response).	Range: 1 to 3.
boutProp	Proportion of the bout of choice sessions (see "total") that the lab condition was chosen (response).	Range: 0 to 1.

Table 2: Variables, their definitions, and the levels or range of the variables for the Choice-based Severity Scale test data (data set 2).

Variables	Definition	Levels/Range
dateTested	Date that the monkey was tested (random effect).	From 2022-04-25 to 2022-05-06
monkeyID	Identity of the monkey being tested (data analyzed separately by monkey).	*monkeyD*: monkey D<br>*monkeyH*: monkey H<br>*monkeyE*: monkey E
sessionNum	Number of the session that the monkey was tested (covariate).	Range: 1 to 10.
trialNum	Number of the trial within a session (covariate).	Range: 1 to 391.
choicePosition	Position of selected option on the touchscreen (factor).	*left*: selected option positioned on the left of the touchscreen.<br>*right*: selected option positioned on the right of the touchscreen.
optionChosen	Option selected by the monkey at the beginning of the trial (response).	*shortHold*: the monkey had to hold a sensor between 1 and 4 s to receive 0.2 to 0.3 ml water reward.<br>*longHold*: the monkey had to hold a sensor between 10 and 40 s to receive 15.2 to 15.3 ml juice reward.

Table 3: Variables, their definitions, and the levels or range of the variables for the condition stimuli training data in preparation for the Choice-based Severity Assessment (data set 3).

Variables	Definition	Levels/Range
dateTested	Date that the monkey was trained (random effect).	From 2020-11-03 to 2021-09-30.
monkeyID	Identity of the monkey being trained (data analyzed separately by monkey).	*monkeyD*: monkey D<br>*monkeyH*: monkey H<br>*monkeyE*: monkey E
sessionNum	Number of the session that the trial type was trained (covariate).	Range: 1 to 27.
trialType	Type of stimuli presented to the monkey (factor).	*upperCage*: upper cage condition stimulus presented simultaneously with a timeout stimulus (for monkey D and H).<br>*lowerCage*: lowe rcage condition stimulus presented simultaneously with a timeout stimulus (for monkey E).<br>*lab*: lab condition stimulus presented simultaneously with a timeout stimulus (all monkeys).
correct	Number of correct trials completed by the monkey (response).	Range: 0 to 39.
total	Total number of trials completed by the monkey (response).	Range: 2 to 44.
